# Manual of fast track recovery for colorectal surgery

**DOI:** 10.1308/003588413X13511609955652

**Published:** 2013-01

**Authors:** Nader Francis, Robin H Kennedy, Olle Ljungqvist, Monty G Mythen

This book brings together everything you need to know about enhanced recovery and more. Its clear layout and concise style enable it to be used both as a manual and as a reference text. It would be a suitable read for all members of the multidisciplinary team.

The title suggests a purely colorectal focus. However, this is misleading as most of the contents refers to the general concept of enhanced recovery and only Chapter 7 focuses purely on colorectal surgery. I would therefore recommend this book as a resource for any surgical specialty looking to implement an enhanced recovery programme.

Despite the extensive reference lists at the end of each chapter, a limitation of this text is that it only includes research up to 2010. Since advances in enhanced recovery are continuous, there are already a number of key studies that have been published in the past two years and are therefore missing from the book. Furthermore, in an era of patient-centred care, perhaps greater focus could have been given to enhanced recovery from the patient’s perspective.

